# Absorbtion

**Published:** 1880-01

**Authors:** James B. Hodgkin

**Affiliations:** D.D.S.


### ABSORBTION.

By JAMES B. HODGKIN, D.D.S.

Of the subject of *absorbtion*, in the broad physiological sense of the books, I shall not of course speak. That of the *bones* of the jaw and of the teeth, as more closely related to dental physiology and pathology, may not prove uninteresting; although, as the subject or the fact itself of absorbtion is the groundwork of nutritive force, one might be tempted into wandering into those pleasant physiological pastures. Cells absorb nutriment matter; the tissues absorb water and fluid plasma, and in a word, the whole complex machine, whether automatic or soul-professed, is dependent on absorbtion. By it we live and move and have our being.

If I call your attention for a short while to some of the curious phenomena, the wonderful play of forces in the growing jaw, I humbly trust I may interest you, for I do not know that anywhere in the body are more strikingly displayed the peculiar action and reaction I have denominated *absorbtion*.

Allow me to freshen you with a few details, which possibly, like all details, if censured, have become indistinct, and drive in your anatomical memories, burdened as they are more or less with clinical study, details that are fresher to us dental students than to strictly medical men. We hunt up our skulls and note some of the anatomical and physiological peculiarities which lie on the surface. We all delight to go back to the beginning of things—it is a strong impulse of the mind to be radical—so we will glance casually at the growth of the fœtal skull. We must begin early in intra-uterine life for this, for as early as the sixth to the ninth week, say the authorities, we may trace the prapillæ of the coming teeth. Lying upon the surface of the mucous membrane of the maxillæ, and gradually enveloped in crypts or lodging places by the growth of the alveolar processes around and over them, they are found at birth quite deeply lodged in the jaw. If I may use the term "inceptment," it will partly represent the condition I wish to express. In those crypts, but divided by septæ, are found not only the gums of the *deciduous*, but these of the *permanent* teeth, all in process of formation, to be erupted in the fullness of time. Calcification here is analogous to ossification of bone.

I call attention to the forms of these teeth. Specially I beg that you will notice that they do not spring forth full-fledged from their bony sockets, but that only the crowns are complete. The roots are entirely lacking, being in this respect a type of the absorbed roots of the temporary teeth, of which mention will be made further on. Nor is this condition of being rootless, or nearly so, incident only to the unerupted condition, for, as you see, the tooth I show you has imperfect roots, and yet it was extracted some seven months after eruption. But more particularly as lying close to the matter under discussion, note that although these roots are so short, and the crowns only are developed, yet we see that the alveolar sockets are fully occupied by them. That is to say, the carities in the bone are no deeper than the prolongation of the teeth downward.

We are describing the *temporary* teeth. Let us start them on their eruptive journey. Advancing, they come by pressure or otherwise, the absorption and disappearance of the alveolar plate above and in contract with them, and emerge from their crypts, taking place in pairs in the line of the jaw. But this, although an interesting phenomenon, is not the only interesting one in this connection, for we see as a coincident of this advance a synchronous advance of the alveolar borders, a movement forward of the whole line, so to speak. And as the emergence of the teeth becomes more and more marked, we see the alveolar borders of their sockets, keeping (in good measure) place with them, until, as the teeth are fully in place, this process arising closes around the cracks of the teeth, holding them in position.

We have thus, in the jawbone itself, a physiological process in three acts. First, The envelopment of the tooth germ in bone; second, The advance of tooth through, and the consequent absorbtion of the roof of its crypt, or so much of it as is necessary for the purpose; and third,

The advance of the process, and investment of the roots by it. Please note the facility with which these changes take place physiologically. Further on we shall see how it is one of the gravest problems in dental pathology and surgery to imitate this physiological act, and induce a re-growth of alveolus where it has been destroyed by a retrograde action, loosening the teeth, and destroying these alveolar sockets.

Just here I should like to call attention to a theory—if it is a mere theory. It seems to be a fact that the alveolar borders are not to be considered as integral parts of the jaws, seeing that they are destroyed, rebuilt, and modified with such facility. The fact that they are mere out-growths, serving their time and office, disappearing when the teeth are lost, and modified by contingencies, would seem below that in the development of the bone they are addenda, so to speak, and not essentials of the maxillæ in their entirety. But I must pass to points which are more directly germain to the subject.

The temporary teeth, ten in number, in each jaw, having taken their places, are in turn made the subjects of one of the most curious and interesting physiological acts in the range of the evolution of the body from puerile to adult life. Again, "in the fullness of time," the growth of the jaw, interstitially and in length, having been considerably advanced, the permanent teeth prepare to take their places in the order in which they are evolved. And please note that with these, as with the temporary set, the growth of the tooth commences *with the crown*, which is first formed, the root following in course of time, and after the eruption of the crown, at least in part. The end of the root is large, opened, truncated, and in appearance entirely unlike finely drawn foramen found in the adult tooth. The dental pulp is seen slightly projecting from the end of the root, and appearing as though it had been cut squarely off.

The upward advance of the tooth, effected by whatever means it may be, (and it is sufficient to say that the old idea of partruition, as applied here, is simply ridiculous), is sufficient to set at work forces, which, acting upon the roots of the temporary teeth, causes their absorbtion, and a removal out of the way of their progress of these hard tissues, so much harder than bone, with a certainty and celerity which had scarcely a parallel in physiological action.

*What is the active agent here?* By what means are these dense, more than osseous structures thus removed? and are we justified in assuming that the absorbed and removed material is used up in the economy? And are we justified in assuming that mere *pressure* is the cause of the destruction or removal of the obstacles in the way of the advance of the permanent teeth? This is the old, and to some extent, still adhered to theory. The advancing crowns of the new teeth were suffered to press upon the roots of the old, and so to cause, in some way, their disappearance. Our present knowledge of the now-vascular structure of the tooth-substance, of course throws out such theories.

If we examine such a tooth as is under discussion we will find, slightly adherent to and in contact with the absorbed surface, a peculiar and highly interesting body—the "carneous body" of Harris, the "cellular mass" of Tonus and others. "The wasting and growing surfaces are readily parted (the wasting tooth and growing cellular mass,) and sometimes this may be effected without hemorrhage, although this "special organ of absorbtion" is highly vascular. Simple contact of surface is all there is here. On examination, histologically this carneous body is seen to consist of "peculiar multiform cells, each composed of smaller cells; the number varying from one to fourteen." Their relation to the wasting dention is peculiar and striking. The various indentations in the inostal surface of the tooth substance are each found to have applied to them as one of the larger cells, each seems engaged in the absorbent act.

Here, then, we have a special organ, engaged in a special act or function, as specially developed as is a plorenta, and as perfectly removed when its work is done. But there are still some facts in connection with its functions which are unsolved, and perplexing. We find this function of absorbtion distinctly intermittent—it progresses, is retarded, is arrested; it retrogrades, it allows new growth, not of dentine proper, for that is never reproduced, but of *cementum*, which is analagous to bone in its structure, and which is deposited in the place of the removed dentine. Again, we must bear in mind that the advancing tooth seems to have little to do with this process of absorbtion, for the temporary teeth are sometimes attacked when no permanent follow to take their places, and thus a vacant space is left; also we see that occasionally the roots of the deciduous teeth are attacked at points, distant from the advancing tooth. And yet again, we see the roots of the permanent teeth themselves sometimes thus wasted away. In advanced age we also see occasionally absorbtion of the roots of teeth and their consequent loss; and finally the roots of teeth which have lost their crowns by decay in many cases, slowly disappear by absorbtion.

I have no pet theory to offer with regard to this physiological act, further than to assert the belief that here, as elsewhere in nature, *special organs are developed for special purposes, which, serving their place and time, are removed;* and to express the probability that the removed lime—salts may not unlikely be used in construction elsewhere—possibly in building the roots of the advancing tooth, or carried into the circulation as plasma.

It is difficult to see why the pathological conditions which arise in first dentition should not recur in the second, especially when we recall the fact that the permanent molars are erupted physiologically as are the temporary teeth, that is, they have no teeth to remove, coming as they do back of the entire first denture. Here the usual course of absorbtion of the alveolus takes place, which in infants creates often grave disturbance, ordinarily without notice.

*The absorbtion of the alveolus* in adults is, as I intimated in the outset, one of the gravest pathological evils with which we, as dentists, have to deal, and before which we stand, if not entirely helpless, at least with grave doubts of mending matters permanently. A disease, ordinarily designated as " Riggis disease," or "compound suppuration of the gums and alveolar processes," consists, briefly, in a gradual averting of the alveolar border, a destruction of the socket of the tooth, and finally its loss. The soundest, firmest teeth are oftenest attacked by this malady, giving rise to an opinion of an incompatibility between the dense tooth substance and the bone adjacent. But it is not proven that the cementum covering the roots of the teeth thus lost is at all more dense than that of other teeth not thus lost, and as no other part of the tooth structure is in contact with the socket, this is seen to be a new hypothesis. A peculiar feature of this absorbtion, is that it is not regular, *i. e.*, it does not progress on all sides of the tooth socket alike. Frequently a molar tooth may be seen in which the lingual root is entirely exposed, the bone gone to the very tip, while the buccal root or roots are firmly attached to the still intact alveolus. That this disease is not secondarily a calcareous deposit is seen from the fact that some of the worst cases are mainly free from deposit. But its etiology is obscure, and with that fact in mind I bring you to the subject, and give you the opinion of an eminent man in your own profession—which, however, comes to me second-hand—that the cause of this destruction of the alveolar border in adult life is due (or was in the case he had under examination) by the administration of mercury in infancy, before the eruption of the temporary teeth. If this be true, it is a grave phase of the mercury subject, and worthy your earnest attention.

The treatment said to be most successful is the thorough removal of the dead border of bone. This is done by suitable scrapers, and the further treatment of the gums and processes by such tonics, astringents, etc., as are deemed suitable. Lately sulphuric acid, dilute or in full strength, is advised, the claim being made that this acid is selective, and will dissolve only the dead bone, and stimulate the living to a healthy growth. Whatever the treatment, it needs to be thorough, and you will appreciate the difficulties dentists labor under in performing such submarine operations. A still newer treatment is very lately suggested—that of removal of the dead bone, the scarification of the soft parts, and their retention in place by a splint of rubber or celluloid. Adhesion is said thus to take place. This is certainly one of the most serious evils which we as dentists have to combat. We arrest decay, or its equivalent; heal and remove abscesses of teeth; cure many of the ills in this direction, and are hopelessly struggling to an adequate treatment of this "absorbtion of the alveolus." Some progress seems to be made, but we are far from satisfied that we thoroughly know either the disease or its remedy.

I have thus briefly laid before you some of the more interesting facts in detail histology and physiology. It is possibly worth while to say that, while the tooth pulp is an active agent in building up tooth substance, it is not known that it possesses the power to pull down, although the studles in this direction are meagre. That the "carneous body," of which I have spoken as the active agent in the destruction of the roots of temporary teeth, should have power not only to remove the hard dentine, but even the almost vitreous enamel, is certainly a wonderful fact, and I have felt that its weight, as a fact possibly somewhat unfamiliar to you, interests you. And I beg that you will bring to the question of the possible action of mercurials administered in infancy, and cropping out in adult life, your best powers of observation in the future.—*Southern Clinic*

